# A Review of Current Knowledge and Guidelines on E-cigarettes: A Healthier Alternative to Classic Cigarettes or Equally Dangerous?

**DOI:** 10.7759/cureus.92685

**Published:** 2025-09-19

**Authors:** Nicole Maryniak, Oliwia Sysło, Nikola Rubik, Izabela Jastrzebska, Weronika Goliat, Maksym Gmur, Konrad Haraziński, Michal Gajewski, Zuzanna Błecha, Alicja Dorota

**Affiliations:** 1 Medicine, Zagłębiowskie Centrum Onkologii Szpital Specjalistyczny im. Sz. Starkiewicza w Dąbrowie Górniczej, Dąbrowa Górnicza, POL; 2 Medicine, Academy of Silesia, Katowice, POL; 3 Internal Medicine, Międzyleski Specialized Hospital in Warsaw, Warsaw, POL; 4 Medicine, Provincial Hospital in Poznań, Poznań, POL; 5 Medicine, Medical University of Silesia in Katowice, Katowice, POL

**Keywords:** asthma, e-cigarette or vaping product use-associated lung injury (evali), electronic cigarettes/e-cigarettes/vaping/e-smoking, electronic nicotine delivery systems, lung cancer

## Abstract

This review was written to summarize the current scientific findings on the safety of e-cigarettes and compare their impact on human disease incidence with that of conventional cigarettes. Due to the significant increase in the popularity of e-cigarettes, especially among adolescents and young adults, we began to wonder whether this form of smoking is actually safer and carries a lower risk of developing and progressing respiratory and circulatory diseases and whether vaping can in some way support users of classic cigarettes in the process of completely quitting smoking. To this end, we reviewed a number of studies published over the years in medical databases, concluding that despite the lower toxicity levels in e-cigarettes, the risk of lung cancer is the same as with conventional cigarettes. The main differences in disease risk occur in cardiovascular and respiratory diseases. E-cigarettes are characterized by a longer time to the onset of symptoms and changes in individual organs, which in turn slows disease progression. Furthermore, replacing conventional cigarettes with electronic cigarettes may reverse some of the changes in the vascular epithelium and reduce the severity of bronchial obstruction in patients with asthma. The fact that e-cigarettes, as a form of nicotine administration, have been developed relatively recently does not allow us to precisely determine the effects of their long-term use and still leaves many doubts that can be dispelled by long-term clinical studies and patient observations.

## Introduction and background

The modern e-cigarette was invented in 2003 by a Chinese pharmacist, Lik Hon, who began working on a healthier alternative to the classic cigarette after his father died of lung cancer. However, work on substitutes has been ongoing since the mid-20th century. During this time, several generations of electronic cigarettes have been created, but it was only the one created in 2003 that gained the favor of a large part of the smoking population. However, the greatest expansion of e-cigarette users occurred in 2008-2009 [1]. Electronic cigarettes owe their popularity primarily to the spread of opinions about the smaller amount of harmful and carcinogenic substances contained in liquids and, consequently, a reduced risk of cancer and respiratory or circulatory diseases with long-term use. Statistical data also show a huge increase in the popularity of this form of nicotine intake among groups of teenagers and young adults: Up to 30% of them regularly used e-cigarettes, and nearly 60% tried it at least once [2]. An additional advantage over classic cigarettes is the multitude of choices of so-called liquids: special oils containing nicotine and various substances that give them attractive, often fruity, sweet flavors, thanks to which users no longer complain about the characteristic unpleasant smell that accompanied the use of classic cigarettes. The common opinion about the greater safety of using e-cigarettes arose due to the lack of scientific research on their harmfulness in long-term use [3]. The impact of substances contained in classic cigarettes has been the subject of numerous clinical studies for decades, and the well-documented impact on health and the risk associated with their long-term use have reached the public opinion and established themselves in the public awareness, while e-cigarettes, as a relatively new method of using nicotine, did not yet allow for tracing its long-term effects on health and the increased risk of specific diseases [4]. The objective of this work is to summarize the current positions of scientific societies and the results of scientific papers that have been developed over the last few years and have allowed for an effective assessment of whether e-cigarettes are actually a healthier alternative to traditional cigarettes and what health effects are associated with their long-term use.

## Review

Safety of use

Due to regulations concerning e-cigarettes, the full composition of substances contained in oils for refilling cartridges is not known; it is also problematic to precisely determine the concentration of nicotine contained in them, and therefore, it is impossible to determine the exact impact and risk associated with the use of substances contained in liquids. As a result of numerous analyses, it was possible to identify some of them and determine what effect their use may have. Nicotine contained in tobacco products leads to strong addiction when used for a long time. As mentioned earlier, we can only determine its approximate content in e-cigarette liquids: Usually, its value ranges from six to 24 mg, but some may contain even over 100 mg of pure nicotine, while classic cigarettes contain approximately 2-8 mg of nicotine in one piece, which gives from 160 mg to 400 mg per pack. On this basis, it can be stated that the average e-cigarette insert contains less nicotine than a pack of classic cigarettes. The real problem, however, is that its exact amount in the liquid is unknown, which carries the risk of overdose and possible toxic or fatal effects [4]. The negative effects of nicotine use mainly concern the circulatory system: it causes the activation of the sympathetic system and, consequently, the activation of beta-adrenergic receptors leading to the increased contractility of the heart muscle, the acceleration of its work, and consequently its greater load. Such long-term, excessive stimulation of the sympathetic system leads to heart failure and greater arrhythmogenic potential.

Additionally, nicotine also stimulates blood vessels, causing them to narrow, which leads to hypertension, and due to reduced blood flow through narrowed vessels, it can lead to the difficult healing of wounds [5]. The substances used as nicotine solvents in e-cigarette liquids are propylene glycol and glycerol: both of these chemical compounds have a negative effect on the respiratory system. Propylene glycol in large doses can cause metabolic acidosis, kidney damage, and even septic conditions. Glycol is added to liquids as a substance that creates clouds of effective smoke coming out of the device during use. However, it can cause episodes of wheezing and a feeling of tightness in the chest, thanks to the activation of receptors involved in the pathogenesis of asthma. Both of these substances also have an irritating effect on the respiratory epithelium, which can cause an exacerbation of an existing disease such as asthma or chronic obstructive pulmonary disease (COPD), as well as increase the risk of respiratory infections [2]. Liquids owe their taste to additives, various sweeteners, and flavorings that give them attractive fruit, sweet, or drink flavors for the user. However, sweeteners such as sucrose and glucose lead to the formation of reactive aldehydes, which significantly contribute to the development of numerous cardiovascular diseases and COPD [6]. The effect of specific e-liquid “flavors” such as menthol, strawberry, or coffee on the vascular endothelium was also examined. Based on the conducted studies, it was concluded that they cause an inflammatory response of endothelial cells and consequently increase the production of pro-inflammatory cytokines such as IL-6 or IL-8. With long-term use, the changes observed in the endothelial cells of people using e-cigarettes were comparable to those presented by users of classic cigarettes [7,8].

Studies have also shown the presence of toxic metals such as chromium, cadmium, and nickel in the fumes released from e-cigarettes during use. It was found that only the concentration of nickel exceeds that contained in the smoke released from classic cigarettes. It has also been proven that the type of device used to heat nicotine affects the content of the abovementioned metals in the mixture of smoke released from the e-cigarette, and so, for example, it was found that e-cigarettes equipped with nickel-chromium atomizers show a significantly greater amount of metals in cigarette smoke than those equipped with stainless steel atomizers. However, it should be remembered that all the metals listed have a carcinogenic effect. The method of inhalation when smoking e-cigarettes and classic cigarettes is very different. When inhaling an aerosol that provides a pleasant taste experience and a feeling of the lack of the irritating effects of tobacco smoke present in classic cigarettes, the inhalation of smoke produced by e-cigarettes is much faster and more frequent and penetrates deeper into the respiratory system, which may entail consequences in the form of the faster development of diseases associated with the regular use of nicotine products. Additionally, it has been proven that, just as in the case of classic cigarettes, the phenomenon of passive smoking occurs, and people staying in the same room with a smoker are exposed to similar side effects as the smokers themselves [9].

Oncogenic effects

As mentioned earlier, the exact oncogenic potential of long-term e-cigarette use is not yet fully understood, and the information that appears in scientific papers concerns only short-term studies, due to the fact that electronic cigarettes are a relatively new method of taking nicotine. Accurate data based on clinical trials are still pending, and their results will not be known until a few to a dozen years from now. However, researchers were able to determine the risk of future non-small cell lung cancer among people who choose e-cigarettes, which will be discussed in the following paragraph.

Lung cancer

Based on the collected clinical studies, it was possible to establish that both e-cigarette and classic cigarette smokers are equally exposed to the occurrence of lung cancer in the future. However, the mechanism of action of e-cigarette aerosol and tobacco smoke from classic cigarettes is different, and it activates different receptor pathways. It has been proven that the aerosol inhaled during the use of electronic cigarettes contributes to the occurrence of non-small cell lung cancer and may also play a role in accelerating the progression of an existing tumor [7,9]. The pathogenesis of non-small cell lung cancer is associated with the activation of a series of reactions by substances contained in e-cigarette aerosol, which have the final effect of reducing the stability of the *DAPK2* gene. *DAPK2* is a tumor suppressor gene belonging to the serine-threonine kinase group; it works by activating the apoptosis of cells suspected of malignant transformation. Decreasing the stability of this gene, and thus disrupting its proper functioning, causes a decrease in the destruction of cancer cells, which gives these cells the opportunity to grow and multiply uncontrolled. The aerosol inhaled during vaping may also contribute to the self-renewal of lung adenocarcinoma cells by inducing SOX2: the nicotine present in the aerosol is mainly responsible for this reaction [9].

Cardiovascular diseases

It has been proven that there is a link between vaping and the risk of cardiovascular incidents among e-cigarette users. The main mechanism of the increase in the risk of such events is related to the action of nicotine, causing the increased activation of the sympathetic nervous system, which was discussed in the paragraph devoted to the action of nicotine itself as a substance. As a result of its action, the heart rate and its workload increase. It has also been proven that substances contained in the aerosol released from electronic cigarettes during inhalation cause an increase in the stiffness of blood vessels, the mechanism of increased oxidative stress, and the depletion of nitric oxide resources, which cause endothelial remodeling and deepen its dysfunction. As a result, there is an increased risk of arterial hypertension and a decrease in blood flow through such changed vessels: this also applies to coronary vessels, in which reduced flow can lead to episodes of myocardial ischemia.

These studies also compared the effect of e-cigarette aerosol to the effect of tobacco smoke from classic cigarettes, and here, rather surprising conclusions were drawn; namely, the effect of activating the sympathetic system and progressive vasoconstriction in e-cigarette smokers was smaller than in classic cigarette smokers. In addition, studies show that vascular changes regressed in smokers who replaced classic cigarettes with electronic ones for a month. On this basis, it was concluded that the use of e-cigarettes may lead to coronary episodes and heart failure in the future, but this risk is lower than in the case of smoking classic cigarettes [10]. Although scientists are already certain that there is a correlation between cardiovascular incidents and vaping, it is currently not possible to precisely determine their risk; for this purpose, more long-term studies are needed that will allow them to be clearly determined.

E-cigarette or vaping product use-associated lung injury (EVALI)

With the emergence of a new method of taking nicotine, a disease entity has also developed, the occurrence of which is closely related to the use of e-cigarettes. The term EVALI itself was introduced relatively recently in 2019 by the CDC and includes symptoms such as cough, chest pain, hemoptysis, and systemic symptoms such as fever or weight loss; in fact, it resembles an acute disease of viral etiology. The common point of all the described cases was the admission of patients to regular and intensive vaping in the 90 days preceding the onset of symptoms. In radiological examinations of patients, a ground glass image, the thickening of interlobular septa, and interstitial inflammatory infiltrates were observed [11,12]. It was noted that the average patient with EVALI is about 24 years old and a White man who is not burdened with chronic diseases but admits to intensive use of e-cigarettes [13]. There is a strong link between the occurrence of EVALI and the use of e-cigarettes, which contain tetrahydrocannabinol (THC) or vitamin E acetate (VEA) in their liquids. Due to the fact that EVALI is a young disease entity, the pathophysiology of its development has not been identified; despite the correlation between the presence of the above compounds in aerosols inhaled during vaping and its occurrence, we currently have relatively little clinical data to clearly determine what mechanisms and transmitter pathways are involved in its pathogenesis [14,15]. For this reason, the diagnosis of EVALI is currently based on a medical history including the occurrence of symptoms, the intensive use of electronic cigarettes in the period immediately preceding the disease, and changes in the radiological image of the lungs [11]. At present, there are no specific guidelines for the treatment of EVALI; in people presenting symptoms, glucocorticosteroids have proven to be helpful so far, but their dosage and duration of use have not been determined. We also do not know the long-term effects of EVALI on the functioning of the respiratory system and possible complications [16,17].

Scientists agree on one thing: more specific research should be carried out to understand the pathogenesis of EVALI and to be able to determine a specific path of treatment and care for patients and perhaps also to limit the occurrence of this disease entity.

Asthma

Due to the huge popularity of e-cigarettes as a way of taking nicotine, many questions have arisen about the degree of exposure of patients with asthma who regularly use electronic cigarettes. Studies and meta-analyses have been conducted that have examined the relationship between smoking e-cigarettes and the risk of developing asthma symptoms and its acute exacerbation [18]. As is already known, the aerosol released from the device during vaporization contains numerous substances that activate the inflammatory cascade in the respiratory tract, thus increasing the release of pro-inflammatory cytokines such as IL-2, IL-6, and IL-8, which also leads to bronchial obstruction, causing the occurrence of asthmatic symptoms. It has also been found that the inflammatory response in people with asthma who smoke electronic cigarettes is greater and stronger, compared to people without a diagnosed disease. A particularly sensitive group at risk of asthma exacerbations is teenagers and young adults, because these groups have seen the largest increase in the number of e-cigarette users compared to other age groups. The results of meta-analyses clearly indicate an increase in reported symptoms such as coughing, the expectoration of phlegm, or episodes of wheezing among both current users and people who have used e-cigarettes in the last month. However, there is no data on the risk of asthma exacerbations during passive exposure to e-cigarette smoke. At the same time, studies show that in people who suffer from asthma but switched from classic cigarettes to electronic cigarettes, the degree of bronchial obstruction and inflammation and the frequency of disease symptoms decreased, so it can be concluded that e-cigarettes contribute to the exacerbation of asthma and the severity of its symptoms, but their impact is smaller than the impact of tobacco smoke inhaled while smoking classic cigarettes [19-21].

## Conclusions

Our findings do not allow us to definitively determine whether the long-term use of e-cigarettes is safer for the user or reduces the risk of disease. Therefore, we cannot say that they are a healthier alternative to traditional cigarettes. However, they possess a number of characteristics that may give them advantages over traditional cigarettes. As we wrote earlier, although the risk of lung cancer and the progression of existing cancer remains the same, in the case of diseases such as hypertension or asthma, switching from traditional cigarettes to e-cigarettes may not only slow the progression of the current disease but also reduce inflammation and reduce the degree of bronchial obstruction. However, it is unknown how this information correlates with the incidence of acute cardiovascular events and the frequency of asthma exacerbations. At the same time, the growing popularity of e-cigarettes among teenagers and young adults has led to the development of a new condition, EVALI. Although its symptoms mimic an acute viral infection, it can ultimately lead to serious respiratory disorders and life-threatening conditions. The effects of EVALI on life and the functioning of the respiratory system are also unknown. It can lead to decreased body function and reduced gas exchange efficiency, leading to chronic hypoxia. Due to the relatively recent boom in e-cigarette use, we cannot predict the effects of long-term nicotine use on users. The answer to this question will likely be known only in a few to a dozen years, when ongoing clinical trials and observations are completed. It is undoubtedly true, however, that for people who used traditional cigarettes and decided to switch to electronic cigarettes, this alternative can provide positive results and slow the progression of certain diseases. E-cigarettes can also prove to be a helpful tool in quitting smoking due to the lower nicotine content of e-liquids.
